# Opioid and Non-Opioid Pharmacotherapy Use for Pain Management Among Privately Insured Pediatric Patients With Cancer in the United States

**DOI:** 10.1093/oncolo/oyad292

**Published:** 2023-11-07

**Authors:** Chan Shen, J Douglas Thornton, Ning Li, Eric Schaefer, Shouhao Zhou, Sarah Kawasaki, Colette Pameijer, Douglas Leslie

**Affiliations:** Department of Surgery, The Pennsylvania State University, College of Medicine, Hershey, PA, USA; Department of Public Health Sciences, The Pennsylvania State University, College of Medicine, Hershey, PA, USA; Department of Pharmaceutical Health Outcomes and Policy, College of Pharmacy, University of Houston, TX, USA; Department of Economics and Finance, Salisbury University, Salisbury, MD, USA; Department of Public Health Sciences, The Pennsylvania State University, College of Medicine, Hershey, PA, USA; Department of Public Health Sciences, The Pennsylvania State University, College of Medicine, Hershey, PA, USA; Department of Psychiatry and Behavioral Health, Penn State Milton S. Hershey Medical Center, Hershey, PA, USA; Department of Surgery, The Pennsylvania State University, College of Medicine, Hershey, PA, USA; Department of Public Health Sciences, The Pennsylvania State University, College of Medicine, Hershey, PA, USA

**Keywords:** pain management, pediatric cancer, opioid and non-opioid pharmacotherapy, observational study, commercial claims data

## Abstract

**Background:**

This study examined the trends and patterns of opioid and non-opioid pharmacotherapy use among a large national sample of privately insured pediatric patients with cancer in the United States.

**Materials and Methods:**

We identified pediatric (aged < 21) patients diagnosed with central nervous system (CNS), lymphoma, gonadal, leukemia, or bone cancer from MarketScan data 2005-2019. We examined the proportion of patients who filled a prescription for the following 5 types of pharmacotherapy: opioid, anticonvulsant, non-steroidal anti-inflammatory drug (NSAID), antidepressant, and muscle relaxant during active cancer treatment. We assessed the trends and patterns in pharmacotherapy using multivariable logistic regressions.

**Results:**

Among 4174 patients included, 2979 (71%) had an opioid prescription; 746 (18%), 384 (9%), 202 (5%), and 169 (4%) had anticonvulsant, NSAID, antidepressant and muscle relaxant prescriptions, respectively. Multivariable logistic regression showed a nonlinear trend in the use of opioids among pediatric patients with cancer over time such that use slightly increased until 2012 (OR of 1.40 [95% CI, 1.12-1.73] for 2012 vs. 2006) but then decreased thereafter (OR of 0.51 [0.37-0.68] for 2018 vs. 2012). The use of anticonvulsants, NSAIDs, and muscle relaxants increased significantly linearly over time (all *P* < .005).

**Conclusion:**

There has been a downward trend in the use of opioids in recent years among pediatric patients with cancer and an upward trend in the use of non-opioid pharmacotherapy for pain management potentially as an alternative to opioids.

**Implications for Practice**

We used a large national database to examine the opioid and non-opioid pharmacotherapy for pain management among 4174 pediatric patients with cancer from 2005 to 2019 and found a downward trend in the use of opioids in recent years among pediatric patients with cancer and an upward trend in the use of non-opioid pharmacotherapy for pain management potentially as an alternative to opioids. Such findings suggest that policy efforts to prevent opioid misuse had impacts on pain management of pediatric patients with cancer and call for future research on the efficacy and potential side effects of non-opioid pain management strategies among patients with pediatric cancer.

## Introduction

Cancer-related pain is a notoriously distressing symptom experienced not only by many adult patients but also by a substantial proportion of the pediatric population with cancer.^1-3^ A study of hospitalized children with cancer suggested that pain was among the most prevalent and bothersome symptoms, being experienced by children in 45% of their hospital days and rated at an average of 4.8 severity score on a 0-10 scale.^3^ To manage pain associated with cancer, opioid medications have long been used as the mainstay.^4,5^ While established evidence documents the efficacy of opioid analgesics in controlling cancer pain,^6-11^ the prevalence of opioid prescriptions leads to many negative consequences, including opioid abuse and misuse, addiction, and overdose that place children and adolescents in a remarkably harmful situation. A recent cross-sectional study of U.S. death certificates showed that between 1999 and 2016, there were an increased number of children dying due to opioid poisonings with the incidence rising from 0.21 to 0.81 per 100 000 children. Furthermore, of the 8986 pediatric deaths due to opioids, 73% involved prescription opioids and the corresponding mortality rate increased by 131.3% from 1999 to 2016.^12^ Other studies also listed opioid prescriptions as one of leading causes for overdoses and misuse in children and adolescents.^13-15^ Combining the substantial need for pain management, the wide use of opioid analgesics, and the potential adverse outcomes among pediatric patients with cancer, it is imperative to study the trends in the use of opioid and alternative pain management pharmacotherapy in pediatric patients with cancer.

In response to the opioid epidemic, many efforts have been devoted to decreasing potentially inappropriate opioid use. Such initiatives include Prescription Drug Monitoring Programs (PDMPs), laws enacted to limit prescribing of opioid medications, CMS opioid policies, provider educational interventions.^16-19^ As regulated and recommended, medical providers are encouraged to consider alternative pain management regimens such as non-opioid pharmacotherapies instead of opioids while achieving satisfactory pain control.^20,21^ This transition from opioid to non-opioid medications would be particularly beneficial for pediatric patients with cancer who are especially susceptible to prolonged opioid use, as their survival rate significantly improves with advancements in cancer treatments.^22,23^ However, little is known for this population with regard to their pain management choices between opioid analgesics and non-opioid agents as well as how such pharmacological management choices are associated with their demographic characteristics. With longer survival and thus more imperative demand for safe and effective pain management among pediatric patients with cancer, there is a critical need to study their opioid and non-opioid pharmacotherapy use.

This study aimed to examine the trends and patterns of opioid and non-opioid pharmacotherapy among privately insured pediatric patients with cancer from 2005 to 2019. We hypothesized a declining trend in opioid use as opposed to an increase in the use of non-opioid therapies during our study period, and patient characteristics and cancer type and treatment would also have large impacts on the choice of pain management approaches.

## Materials and Methods

### Data Source

The data source for this observational study is the IBM Truven MarketScan Commercial Claims and Encounters Database. This database is a large nationwide database based on private insurance claims covering 50 million unique patients enrolled in various commercial health insurance plans including health maintenance organizations (HMOs), preferred provider organizations (PPOs), point-of-service (POS) plans, and indemnity plans and a variety of coverage, such as privately insured fee-for-service, POS, or capitated health plans. It provides detailed information on patient demographic characteristics, insurance enrollment information, and claims information, which can be used to capture inpatient, outpatient, and prescription drug usage. It is a well-accepted data source for health services and outcomes research. We included data from MarketScan up to the end of 2019, which was the most recent year of data available at the time of this study.

### Study Cohort

We identified pediatric patients with cancer less than 21 years of age, diagnosed with one of the following 5 cancer types most common in this age range: central nervous system (ie, CNS including brain and spinal cord), lymphoma, gonadal, leukemia, or bone cancer from January 2005 to December 2018 based on the International Classification of Diseases, Ninth and Tenth Revisions (ie, ICD-9 and ICD-10) diagnosis codes based on Agency for Healthcare Research and Quality (AHRQ) Clinical Classifications Software codes.^24,25^ Specifically, we required that the patients had at least 2 cancer-related visits based on the diagnosis codes. We captured pediatric cancer treatments including chemotherapy, surgery, and radiation based on Current Procedural Terminology (CPT) codes. We identified the initial cancer diagnosis date, ensuring that there were no other cancer-related claims in the 6 months preceding this diagnosis. The end of the treatment date was determined as 30 days following the last observed treatment claim. We stipulated that patients should have no further cancer treatment claims for 1 year after this end-of-treatment date. The end-of-treatment date is required to be prior to when the patient turns 21 years of age. The period spanning from the initial cancer diagnosis to the end-of-treatment date is defined as the active cancer treatment phase. To guarantee the completeness of records for determining the start and end of active treatment, we required that patients have continuous enrollment in an insurance plan from 6 months before the diagnosis date to 1 year post the end-of-treatment date.

### Use of Opioid and Non-Opioid Pharmacotherapy

We identified the use of opioid and non-opioid pharmacotherapy for pain management based on National Drug Code (NDC). We considered the following 4 types of non-opioid pharmacotherapy with evidence for use to manage chronic pain: anticonvulsant, NSAID, antidepressant, and muscle relaxant.^26^ There is a growing literature on the use of these non-opioid pain management pharmacotherapies in patients with cancer, although evidence on the efficacy of them among pediatric patients with cancer is still scarce.^27-30^

### Statistical Analyses

We reported sample descriptive statistics using frequency counts and percentages for our study cohort. We provided figures demonstrating the percentages of patients using each pharmacotherapy over time by cancer site. We conducted multivariable logistic regression for each type of pharmacotherapy for pain management. The outcome variable is a binary variable indicating whether the patient received the respective pharmacotherapy for that model (ie, opioid, anticonvulsant, NSAID, antidepressant, or muscle relaxant). The explanatory variables included age, sex, year of cancer diagnosis, cancer treatment received (3 binary variables indicating whether surgery, chemotherapy, and radiation were received), cancer site (CNS, lymphoma, gonadal, leukemia, or bone) and time in active cancer treatment. We note that we included year of cancer diagnosis as a linear effect in all models with the exception of the model for opioid use, which was highly non-linear. In that case, a b-spline with 3 degrees of freedom was used. The use of linear and flexible b-spline effectively reduces the number of parameters in contrast to incorporating multiple indicators for the year of diagnosis. This approach not only mitigates potential multicollinearity concerns but also ensures the necessary statistical power with the fixed sample size,^31,32^ allowing us to capture the key time trends in the data without overfitting. The adjusted odds ratios (ORs), *P*-values and 95% confidence intervals (95% CIs) were reported.

All statistical analyses were conducted in SAS 9.4 (SAS Institute, Cary, NC) and R 3.6.0 (R Core Team, Vienna, Austria). This study was exempted by Institutional Review Board at Penn State College of Medicine, as all patients in the database had been de-identified.

## Results

The study included 4174 pediatric patients with cancer first diagnosed with CNS, lymphoma, gonadal, leukemia, or bone cancer in 2005-2018. The detailed sample descriptive characteristics are provided in Table 1. The median age at cancer diagnosis was 15 with IQR of 8-18; while the median age at the end of cancer treatment was 15 with IQR of 9-19. The majority (67%) of the patients received surgery, close to a half (48%) received chemotherapy, and approximately a quarter (26%) received radiation therapy for cancer treatment. We found that the vast majority (71%) received opioids, 18% received anticonvulsant, 9% received NSAIDs, 5% received antidepressants, and 4% received muscle relaxant.

**Table 1.** Patient and treatment characteristics stratified by year of index date.

	2005-2009 (*N* = 1305)	2010-2013 (*N* = 1533)	2014-2018 (*N* = 1336)	Total (*N* = 4174)	*P*-value
Age, median (IQR)	14 (7-17)	15 (8-18)	15 (9-19)	15 (8-18)	<.001
Male gender, *N* (%)	755 (58)	913 (60)	757 (57)	2425 (58)	.29
Region, *N* (%)					<.001
Northeast	149 (11)	244 (16)	249 (19)	642 (15)	
North central	360 (28)	411 (27)	301 (23)	1072 (26)	
South	561 (43)	547 (36)	547 (41)	1655 (40)	
West	229 (18)	307 (20)	227 (17)	763 (18)	
Unknown	6 (1)	24 (2)	12 (1)	42 (1)	
Type of cancer, *N* (%)					.011
CNS	430 (33)	466 (30)	400 (30)	1296 (31)	
Lymphoma	268 (21)	331 (22)	338 (25)	937 (22)	
Gonadal	180 (14)	258 (17)	213 (16)	651 (16)	
Leukemia	217 (17)	249 (16)	180 (14)	646 (16)	
Bone	210 (16)	229 (15)	205 (15)	644 (15)	
Surgery, *N* (%)	872 (67)	1025 (67)	915 (69)	2812 (67)	.57
Chemotherapy, *N* (%)	574 (44)	699 (46)	723 (54)	1996 (48)	<.001
Radiation, *N* (%)	401 (31)	419 (27)	270 (20)	1090 (26)	<.001
Age at end of treatment, median (IQR)	15 (8-18)	15 (9-19)	16 (10-19)	15 (9-19)	<.001
Months of treatment, median (IQR)	4.5 (1.6-10.4)	4.5 (1.4-9.5)	4.4 (1.3-8.2)	4.5 (1.4-9.2)	.013
Opioid during treatment, *N* (%)	912 (70)	1129 (74)	938 (70)	2979 (71)	.046
Anticonvulsant during treatment, *N* (%)	183 (14)	270 (18)	293 (22)	746 (18)	<.001
NSAID during treatment, *N* (%)	107 (8)	134 (9)	143 (11)	384 (9)	.06
Antidepressant during treatment, *N* (%)	60 (5)	72 (5)	70 (5)	202 (5)	.71
Muscle relaxant during treatment, *N* (%)	38 (3)	52 (3)	79 (6)	169 (4)	<.001

**Figure 1.**
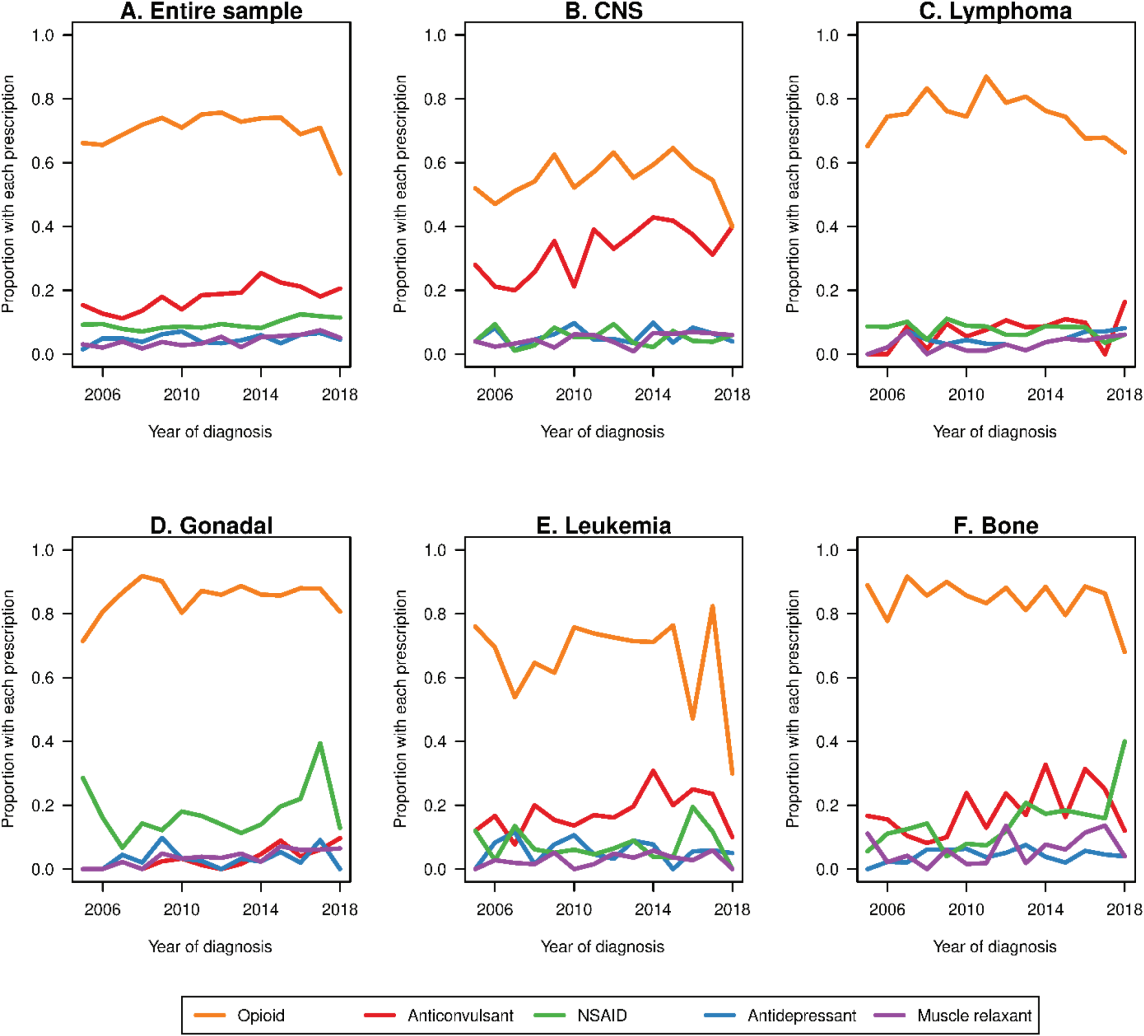
Proportions of patients with each type of prescription during the treatment period for the entire sample (**A**) and stratified by cancer type (CNS [**B**], lymphoma [**C**], gonadal [**D**], leukemia [**E**], and bone [**F**]).

**Figure 2.**
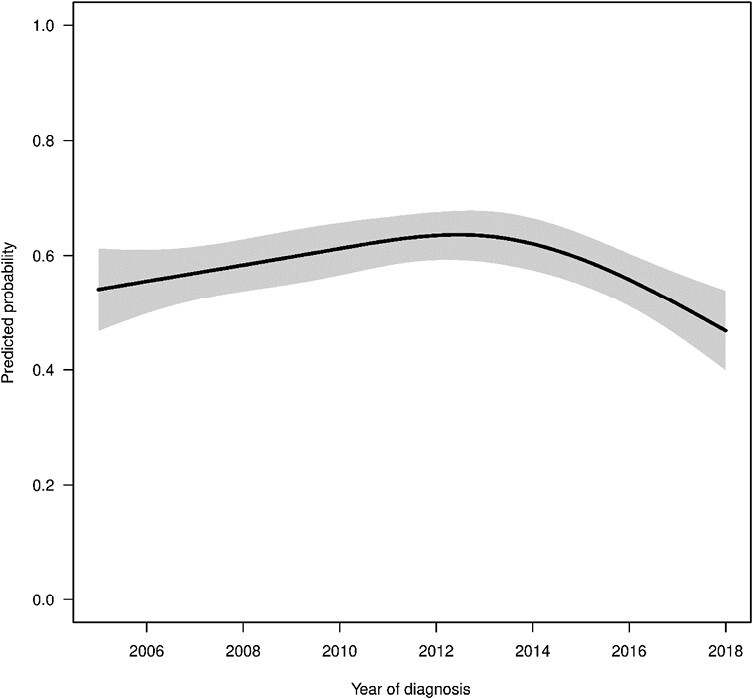
Predicted probabilities of patients filling a prescription for opioids by year of diagnosis estimated from multivariable regression model. All other variables in the model were set to the median (continuous variables) or most common response (categorical variables) for this figure.

Fig. 1 provides a visual representation of the time trends in the use of opioid and non-opioid pharmacotherapy over the study period with year of cancer diagnosis on x-axis and proportion of patients with each pharmacotherapy on the y-axis. We provided the trends for the entire sample, and also for each cancer type. We note that the subplots in Fig. 1 exhibit considerable fluctuations potentially due to the limited sample size after splitting by cancer type and year of diagnosis. Although Fig. 1 showcases raw frequencies categorized by cancer types and years, it primarily offers an unadjusted view of the trends without considering vital patient characteristics, such as age, sex, types of cancer treatments (surgery, radiation, and chemotherapy), and duration of active treatment. Our multivariable logistic regression (Table 2) and its corresponding figure (Fig. 2), which account for these factors, provide a better depiction of opioid usage trends over time. From the figure, we observe that the use of opioids decreased over the study period with patients with lymphoma showing the most pronounced decrease in opioid use dropping from above 80% to around 60%; the use of anticonvulsants increased during this time with the most pronounced increasing trend observed in patients with CNS from around 20% to 40%; and the use of NSAIDs also increased with patients with bone cancer showing the largest increase from less than 10% to around 40%.

**Table 2.** Odds ratios from logistic regression models for each type of prescription during treatment period.

Outcome	Parameter	OR (95% CI)	*P*-value
	Age, 1-year increase	1.08 (1.07-1.10)	<.001
Opioid	Sex (female vs. male)	1.22 (1.05-1.41)	.009
	Year of diagnosis (non-linear)		<.001
	2012 vs. 2006	1.40 (1.12-1.73)	.003
	2018 vs. 2012	0.51 (0.37-0.68)	<.001
	Surgery	1.50 (1.22-1.84)	<.001
	Chemotherapy	1.50 (1.23-1.83)	<.001
	Radiation	1.17 (0.98-1.40)	.09
	Type of cancer		
	CNS (ref)	1	
	Lymphoma	1.46 (1.17-1.83)	.001
	Gonadal	3.41 (2.59-4.48)	<.001
	Leukemia	1.34 (1.01-1.78)	.048
	Bone	3.93 (3.06-5.05)	<.001
	Time in treatment period, 1-year increase	1.51 (1.36-1.67)	<.001
	Age, 1-year increase	1.08 (1.06-1.10)	<.001
**Anticonvulsant**	Sex (Female vs. male)	1.27 (1.07-1.51)	.006
	Year of diagnosis, 1-year increase	1.08 (1.06-1.11)	<.001
	Surgery	1.88 (1.44-2.44)	<.001
	Chemotherapy	1.25 (0.99-1.59)	.06
	Radiation	0.87 (0.71-1.07)	.18
	Type of cancer		
	CNS (ref)	1	
	Lymphoma	0.09 (0.07-0.13)	<.001
	Gonadal	0.04 (0.02-0.06)	<.001
	Leukemia	0.48 (0.34-0.68)	<.001
	Bone	0.35 (0.27-0.44)	<.001
	Time in treatment period, 1-year increase	1.37 (1.26-1.49)	<.001
	Age, 1-year increase	1.12 (1.09-1.15)	<.001
**NSAID**	Sex (female vs. male)	1.78 (1.42-2.24)	<.001
	Year of diagnosis, 1-year increase	1.05 (1.02-1.08)	.004
	Surgery	0.87 (0.63-1.19)	.38
	Chemotherapy	0.81 (0.62-1.06)	.13
	Radiation	0.94 (0.70-1.25)	.65
	Type of cancer		
	CNS (ref)	1	
	Lymphoma	1.09 (0.74-1.62)	.66
	Gonadal	3.12 (2.14-4.56)	<.001
	Leukemia	1.01 (0.62-1.64)	.98
	Bone	2.49 (1.76-3.53)	<.001
	Time in treatment period, 1-year increase	1.73 (1.56-1.92)	<.001
	Age, 1-year increase	1.22 (1.17-1.27)	<.001
**Antidepressant**	Sex (female vs. male)	1.87 (1.39-2.52)	<.001
	Year of diagnosis, 1-year increase	1.01 (0.97-1.06)	.54
	Surgery	1.10 (0.73-1.46)	.65
	Chemotherapy	1.58 (1.08-2.32)	.019
	Radiation	1.03 (0.73-1.46)	.85
	Type of cancer		
	CNS (ref)	1	
	Lymphoma	0.28 (0.17-0.44)	<.001
	Gonadal	0.26 (0.15-0.46)	<.001
	Leukemia	0.81 (0.47-1.40)	.44
	Bone	0.51 (0.32-0.82)	.005
	Time in treatment period, 1-year increase	1.55 (1.37-1.76)	<.001
	Age, 1-year increase	1.20 (1.15-1.25)	<.001
**Muscle relaxant**	Sex (Female vs. male)	1.17 (0.84-1.62)	.35
	Year of diagnosis, 1-year increase	1.09 (1.04-1.14)	.001
	Surgery	1.81 (1.13-2.88)	.013
	Chemotherapy	2.77 (1.86-4.14)	<.001
	Radiation	0.98 (0.67-1.44)	.93
	Type of cancer		
	CNS (ref)	1	
	Lymphoma	0.18 (0.10-0.31)	<.001
	Gonadal	0.27 (0.15-0.48)	<.001
	Leukemia	0.43 (0.22-0.86)	.016
	Bone	0.76 (0.49-1.19)	.24
	Time in treatment period, 1-year increase	1.52 (1.32-1.75)	<.001

The multivariable logistic regression results are provided in Table 2. The logistic regression showed that opioid use significantly increased until approximately 2012 and then decreased substantially thereafter. For example, the adjusted OR of opioid use comparing 2012 with 2006 was 1.40 (95% CI, 1.12-1.73, *P*-value = .003), while the OR comparing 2018 with 2012 was 0.51 (95% CI, 0.37-0.68, *P*-value < .001). To better illustrate the nonlinear trend in opioid use, we provided Fig. 2 which shows the predicted probabilities of patients filling a prescription for opioids by year of diagnosis estimated from multivariable regression model keeping all other variables at either median (continuous variables) or most common response (categorical variables). The type of cancer had strong association with opioid use. For example, the OR for patients with bone cancer compared to patients with CNS was 3.93 (95% CI, 3.06-5.05, *P*-value < .001). The use of surgery and chemotherapy was significantly associated with more opioid use (OR = 1.50, 95% CI, 1.22-1.84, *P*-value < .001 and OR = 1.50, 95% CI, 1.23-1.83, *P*-value < .001 respectively), while radiation was not significantly associated with opioid use. Furthermore, the time treatment period was also significantly associated with higher likelihood of receiving opioids (OR = 1.51, 95% CI, 1.36-1.67, *P*-value < .001). We found that older age (per 1-year increase in age: OR = 1.08, 95% CI, 1.07-1.10, *P*-value < .001) and female sex (OR = 1.22, 95% CI, 1.05-1.41, *P*-value = .009) were significantly associated more opioid use.

The multivariable logistic regression for use of anticonvulsant showed a significant increase by year of cancer diagnosis with OR of 1.08 (95% CI, 1.06-1.11, *P*-value < .001) per year. Patients with CNS cancer were significantly more likely to use anticonvulsants. The ORs of anticonvulsant use comparing other cancer types to CNS were all less than 0.5 (*P*-value < .001). Similar to the use of opioids, older age, female sex, surgery, and time in treatment were also significantly associated with anticonvulsant use.

The results from the multivariable logistic regression for NSAID use also showed a significant increase over the study period (OR = 1.05, 95% CI, 1.02-1.08, *P*-value = .004). Patients with gonadal and bone cancer were significantly more likely to receive NSAIDs compared to CNS (OR = 3.12, 95% CI, 2.14-4.56, *P*-value < .001 and OR = 2.49, 95% CI, 1.76-3.53, *P*-value < .001, respectively). Older age, female sex, and longer time in treatment were again significantly associated with NSAID use (all *P*-values < .001).

It is interesting that the logistic regression did not show significant increase in the use of antidepressants over the study period (*P* = .54), while the multivariable logistic regression for muscle relaxant found significant increase in the use of muscle relaxant during the study period (1-year increase: OR = 1.09, 95% CI, 1.04-1.14, *P*-value = .001).

## Discussion

This paper is the first comprehensive study of pain management strategies for pediatric patients with cancer. We conducted analyses of the trends and patterns of opioid and non-opioid pharmacotherapies for a privately insured pediatric population diagnosed with cancer between 2005 and 2019. The results suggested that the utilization of opioid analgesics increased up to 2012 and then fell sharply, while non-opioid medication use rose over time. Patient age, cancer type, and treatment were found to be significantly associated with the choice of pain management approaches. With few recent large observational studies on the use of pharmacotherapies in pediatric cancer population,^33-35^ this paper contributes substantially by providing a thorough investigation of the current use of pharmacological pain management in this particularly vulnerable patient group using a large national database, which may inform the evaluation and development of public health measures and initiatives designed to contain the unprecedented opioid epidemic in the United States.

Opioid use is highly prevalent in the setting of cancer care for both adult and pediatric patients. As the first-line analgesics, opioids are commonly prescribed to patients in managing their cancer-related pain. Prior literature found that strong opioids were prescribed to effectively control pain in approximately three-quarters of patients with cancer.^6,7,36^ An observational study of the US data reported that 77.7% of pediatric patients newly diagnosed with acute myeloid leukemia were exposed to opioids in the course of treatment.^37^ In this study, we also found a majority of pediatric patients with cancer (>70%) received opioid prescriptions.

More importantly, we found an increase from 2006 to 2012 and then a sharp decrease in opioid use from 2012 to 2018 among pediatric patients with cancer diagnosed in these years. This trend is perfectly in line with national overall prescription opioid dispensing rate over the same time period.^38^ Specifically, a CDC report showed a steady increase in the overall national opioid dispensing rate starting in 2006, which peaked in 2012 at 81.3 prescriptions per 100 persons, and then a substantial decline from 2012 to 2020, which has decreased to 43.3 prescriptions per 100 persons in 2020.^38^ Our findings suggest that the many efforts to prevent inappropriate opioid use starting around year 2012 such as state regulations on pain clinics and implementation of PDMPs (eg, 2010 in Florida, 2011 and 2012 in Ohio and Kentucky, 2012 in New York and Tennessee),^39^ federal rescheduling of hydrocodone in 2014,^40^ many state opioid dosing guidelines and provider educational interventions^41^ may have raised awareness of potential harms of prescription opioids in the pediatric cancer care community and reduced opioid prescriptions in this particular population. Even though most policy efforts are mainly focused on the adult non-cancer population,^12,35^ such efforts may have elicited spillover effects on physician attitudes toward opioids in other patient groups such as pediatric patients with cancer.

We also found a significant increase in non-opioid pharmacotherapy use over time in pediatric patients with cancer. Similar utilization trends were also revealed in the literature, with recent analysis of a cohort of patients with acute myeloid leukemia from the Pediatric Health Information System suggesting a rise in non-opioid utilization from 70% to 88.2% from 2000 to 2014.^37^ Our study confirmed the concurrent growth in non-opioid pharmacotherapy use and decrease in opioid use in recent years. The finding of growing non-opioid pharmacotherapies calls for more research including clinical trials on the efficacy and potential side effects of non-opioid pain management strategies among pediatric patients with cancer.

In addition, our study showed that the time trends in the use of opioid and non-opioid treatments varied with the type of cancer. Out of the 5 types of cancer investigated in this study, patients with lymphoma showed the most pronounced decrease in opioid use dropping from above 80% to around 60%. Patients with bone cancer showed the largest increase in the use of NSAIDs from less than 10% to around 40%. Patients with CNS experienced the most pronounced increasing trend in the use of anticonvulsants from around 20% to 40%. Although there have been research showing the pain management benefit of anticonvulsants in patients with cancer,^27-30^ anticonvulsants are also well-established as evidence-based drugs to manage epilepsy in patients with brain tumor and therefore widely used.^42-44^ Therefore, the increasing use of anticonvulsants in patients with CNS may be related to not only decrease in opioid use but also the management of other conditions than pain.

The type of cancer was also an important factor associated with pain management strategy. Compared to patients with CNS, gonadal and bone cancer were associated with more opioid use. It is well known that cancer pain is associated with the type of cancer, and bone cancer is one of the most painful cancers. Therefore, it is not surprising that patients with bone cancer were more likely to have opioid prescriptions. The result is also in line with another study mainly focused on pharmacotherapy 1 to 3 years after active treatment among pediatric survivors with cancer that also documented the higher rate of opioid use among patients diagnosed with bone and gonadal cancer than those with CNS cancer.^23^ We also observed varying preferences for non-opioid medication across different cancer types. For instance, the administration of anticonvulsants and antidepressants to patients with CNS cancer was greater than to other cancer types. This is probably driven by the fact that patients with CNS cancer commonly experience seizures,^45^ and anticonvulsants are recommended as effective antiepileptic drugs with the additional benefit of pain relief.^46,47^ Furthermore, cancer diagnosis and treatment, uncontrolled seizures, and side effects of anticonvulsant use are also factors associated with an elevated risk of mental disorders,^48-50^ including depression, leading to the higher use of antidepressants among patients with brain cancer. Conversely, patients with gonadal cancer had higher tendency to be prescribed NSAIDs than those with CNS cancer. This inclination toward NSAIDs in pediatric patients with gonadal cancer aligns with evidence suggesting the benefits of NSAIDs for adult patients with reproductive system cancers. Notably, aspirin use has been documented to be associated with improved prognosis of patients with ovarian cancer,^51^ and the high-intensity use of non-aspirin NSAIDs is found to lower mortality of patients with serous ovarian cancer.^52^ Patients with bone cancer also received NSAIDs more frequently than patients with CNS cancer. Similarly, this is in line with the enhanced pain management efficacy and fewer side effects of NSAIDs combined with opioids in adult patients with bone cancer.^53^

Opioid use was positively correlated with age, which was consistent with a majority of prior studies.^21,37,54^ We also found that female sex was significantly associated with more opioid use. Mixed evidence was suggested in the literature on the opioid exposure by gender in children with cancer. While one study showed that female children with cancer were more likely to be exposed to opioids,^54^ another study indicated that the difference in the prevalence of opioid exposure between female and male pediatric patients with cancer was statistically insignificant.^37^ The latter also suggested that once exposed, female patients experienced longer duration of opioid use. In addition, undergoing oncological surgery and chemotherapy were also correlated with more opioid use, which might imply more severe pain due to invasive procedures that needed strong analgesics. Non-opioid use was also positively associated with age and female sex. In particularly, female patients had significant higher odds than male patients of using antidepressants, which aligned with the higher prevalence of depression among females than males and the concomitant gender disparity in antidepressant use.^55,56^

This study focused on the pain management during active treatment phase of pediatric cancer, distinct from another body of literature that delves into the pediatric cancer survivorship post-treatment.^22,23^ To investigate the post-treatment pharmacotherapy prescriptions, one needs to follow patients considerably longer post-treatment, similar to the approach taken by Smitherman et al, which examined 1414 patients who completed cancer treatments between 2000 and 2011.^23^ As newer data emerges, a promising avenue for future research might be to reassess prescription drug usage patterns during both the treatment and post-treatment phases among pediatric patients with cancer in subsequent years and also to explore the potential impact of the COVID-19 pandemic on pain management of pediatric patients with cancer.

Our study has some limitations. First, the data do not include information on the pain type, severity, or duration of patients. Therefore, we do not know the reason for which the pharmacotherapies were prescribed and how well controlled the pain was as the use of opioids decreased and of non-opioids increased. Lack of pain data may also mask the reason of the disparity in utilization patterns by characteristics, given the possibility that a subgroup is engaged in more use in response to their higher prevalence of severe pain than other subgroups. Further investigation of the outcome of pain management is needed in further research. Second, the database only includes claims for prescription drugs, and therefore, over-the-counter drugs such as lower-strength NSAIDs may not be captured. Third, for some of the non-opioid pharmacotherapy, there is a chance that these medications were prescribed for other conditions not primarily related to pain management. Finally, claims data only provide records of filling prescriptions but do not necessarily indicate the true utilization of those prescription medications, which may lead to inaccuracies with regard to the actual use of opioid and non-opioid pharmacotherapy in this study.

## Conclusions

In this large observational study of 4174 privately insured pediatric patients with cancer during cancer treatment, we found a significantly decreasing trend in the use of opioid prescriptions over the study period since 2012. Meanwhile, there was an upward trend in the use of other pharmacotherapies such as NSAIDs and anticonvulsants. Furthermore, we found large variations in pain management strategies by age, sex, type of cancer, and cancer treatment received. Further research is warranted to determine those pain management strategies for pediatric patients with cancer that provide efficacious degrees of analgesia with minimization of drug side effects.

## Data Availability

The data underlying this article were provided by MarketScan under licence/by permission. Data will be shared on request to the corresponding author with permission of MarketScan.
